# “It’s not about a question, it’s about the outcomes, isn’t it?”: pilot study for Scottish pregnancy screening tool provision of preconception health care in Scotland

**DOI:** 10.1186/s12978-025-02191-y

**Published:** 2025-11-21

**Authors:** Emma J. Brough, M. Adília Lemos, Karen L. Barton

**Affiliations:** Faculty of Social and Applied Sciences, Abertay University, Dundee, Scotland, UK

**Keywords:** Preconception, Pregnancy intention, Pregnancy screening, Preconception intervention, Pregnancy prevention, Preconception care

## Abstract

**Background:**

Pregnancy intention is not routinely asked about in most primary care settings, leading to missed opportunities for timely discussions about contraception or preconception care. This study evaluated a Scottish adaptation of the One Key Question^®^ (OKQ^®^) screening tool, designed to prompt conversations about pregnancy desire with women of reproductive age.

**Methods:**

The pilot was implemented in two GP practices in Scotland. All women aged 16 and over with the capacity to become pregnant were eligible to be asked the question, regardless of the reason for their visit. Information, services, or referrals were then offered based on their response. Quantitative and qualitative methods were used to evaluate acceptability from the perspective of patients and healthcare professionals (HCPs) over a 3-month period.

**Results:**

Fifty-six women were asked the pregnancy intention question from October to December 2024. Thirty-eight (68%) provided feedback. Most found the conversation easy (92%) and felt listened to and supported (94%). Six HCPs were interviewed at the end of the pilot; they described the tool as a useful prompt, with contraception the most common outcome. However, some reported uncertainty about when it was appropriate to ask.

**Conclusion:**

This pilot suggests that using a standardised pregnancy intention question may be a simple and acceptable way to initiate reproductive health conversations in routine care. The findings also highlight the need for flexible and sensitive use depending on patient context.

**Supplementary Information:**

The online version contains supplementary material available at 10.1186/s12978-025-02191-y.

## Abstract (plain language summary)

Looking after health before becoming pregnant is important for improving outcomes for both mothers and babies. However, pregnancy intentions are not routinely discussed in primary care in Scotland, and this can mean missed opportunities to support women who are planning a pregnancy or who do not want to become pregnant.

This study tested a pregnancy screening tool in two GP practices in Scotland. The tool asked women of childbearing age about their pregnancy intentions and supported healthcare professionals to start conversations about reproductive health.

Over a three-month period, 56 women were asked about their pregnancy intention. Most found it acceptable, and healthcare professionals reported that it led to useful discussions, particularly around contraception and medication safety.

The study shows that this type of screening tool can be used in routine primary care to support open conversations about pregnancy planning and reproductive health. Further research is needed to explore the best ways of using the tool consistently in practice.

**Supplementary Information:**

The online version contains supplementary material available at 10.1186/s12978-025-02191-y.

## Background

Preconception health refers to the health status and risk factors of individuals before conception occurs. It plays a crucial role in improving pregnancy outcomes, reducing maternal and infant morbidity and mortality, and promoting long-term health for both parents and children [1] ​. Globally, poor preconception health due to factors such as obesity, poor diet, smoking, alcohol and drug use and mental health issues, leads to adverse pregnancy outcomes. These risks are often linked to social and economic deprivation [2]​. A previous study argues that pregnancy planning, and preparation remains more of a concept than a reality due to policy challenges that make preconception health difficult to differentiate from extensive public health objectives around healthy lifestyles and that a person’s childbearing years are a considerable period in the life span [3]​. Preconception health optimisation is a continuous process rather than a one-time intervention. A life course approach to preconception health, which integrates pregnancy prevention and preparation, is essential. Policy development is challenged by the difficulty of delineating preconception health from broader population health priorities. The reproductive years span a significant portion of the life course, yet the period during which an individual is actively trying to conceive or is pregnant is relatively short. This makes preconception health a critical window of opportunity—but one that often slips between the stools of public health planning, falling outside the clear remit of existing services and strategies. As a result, targeted interventions risk being either too narrow to be impactful or too broad to be operationally distinct. Scotland is currently developing a Population Health Framework with a focus on prevention and early intervention [4]​. It is essential that this framework explicitly includes the preconception period, given the persistent and widening health inequalities across the country. A recent population-based study analysing national data over a 15-year period found an increase in preterm births that could not be fully explained by maternal age or socioeconomic status—suggesting that other modifiable, and potentially preventable, risk factors are at play [5]​.​ These findings highlight the critical importance of optimising health before pregnancy begins. Adverse birth outcomes are closely linked to poverty and economic stressors, and previous research [6]​ further demonstrates that economic policies influencing income and access to social services can have a profound effect on maternal and infant health. Addressing these wider social determinants is therefore not only essential for improving preconception health but also for reducing entrenched health inequalities across the life course.

There is currently no data from Scotland about pregnancy intention screening. The most recent Public Health Scotland report on terminations shows that Scotland’s average termination rate was 17.6 per 1,000 women aged 15–44 in 2023. This report from 2023 shows that terminations in Scotland have increased by 10% (1,600) from 16,607 in 2022 to 18,207 in 2023 [7]​​. Demographics of the pregnant population in Scotland have changed but this increase was observed across all age groups. There was an obvious difference in termination rates between women living in the most (24.1 per 1,000) and least deprived areas (12.4 per 1,000), highlighting the possible inequalities in access to contraception for women living in more deprived areas [8]​​. It is not known how many of the 44,383 births in 2023/24 were unplanned, ​ but it is known that the health of a pregnant woman and her baby are intricately linked, this includes health prior to conception [9]. There is also more recent, emerging data on the importance of men’s health in contributing to the health of the foetus [10]. 

*One Key Question* (OKQ)^®^ is currently a programme of Power to Decide, a non-profit organisation, and was the inspiration for this Scottish adaptation of a reproductive screening tool [11]. OKQ^®^ was originally created and developed by the Oregon Foundation for Reproductive Health to screen women for their reproductive health services need, whether this be contraception, preconception, or maternal health advice [12, 13]​. Most data from OKQ^®^ comes from the USA. There have been several evaluations on this screening tool showing that it is effective in having a positive impact on reproductive care [14–16]. In addition, a previous study showed that asking about pregnancy intention in primary care is acceptable and feasible [17].

Due to growing health inequalities across Scotland and the rest of the UK, there is a growing recognition of the need for consistent patient- centred approaches to reproductive health. Universal screening tools, such as the adapted OKQ^®^, may offer a practical way to initiate conversations about pregnancy desire within routine healthcare visits [17]. The Scottish adaptation of OKQ^®^ is an approach that allows an accommodating and empowering conversation to take place between a patient and Health Care Practitioners (HCPs) during routine visits. The screening tool aligns with calls from Stephenson et al. [18] to normalise preconception conversations within healthcare, but it is essential that these discussions are implemented sensitively and contextually avoiding overuse or the implication that women are defined by their reproductive status. This approach may also help HCPs to recognise patients who are at risk of unplanned pregnancy or who desire a pregnancy and would benefit from timely preconception health information and care. The pregnancy screening tool was designed to be a patient-centred method for initiating these conversations. The aim of this evaluation was to assess the acceptability and usability of this pregnancy screening tool from the perspective of patients and HCPs in a Scottish clinic setting. The evaluation was divided into three components: how women attending the clinics perceived the pregnancy desire question, the service delivery issues and outcomes identified by healthcare providers (HCPs), and how the HCPs themselves perceived the experience of asking the question.

## Methods

Two Scottish primary care clinics (one in Glasgow and the other in Edinburgh) conducted a pilot study from October to December 2024 to assess the acceptability and perceived impact of a pregnancy intention screening tool, explore how it was integrated into routine consultations, and identify factors influencing its use. The description and details of the methods used are described below.

### Setting and participants

**Fig. 1:**
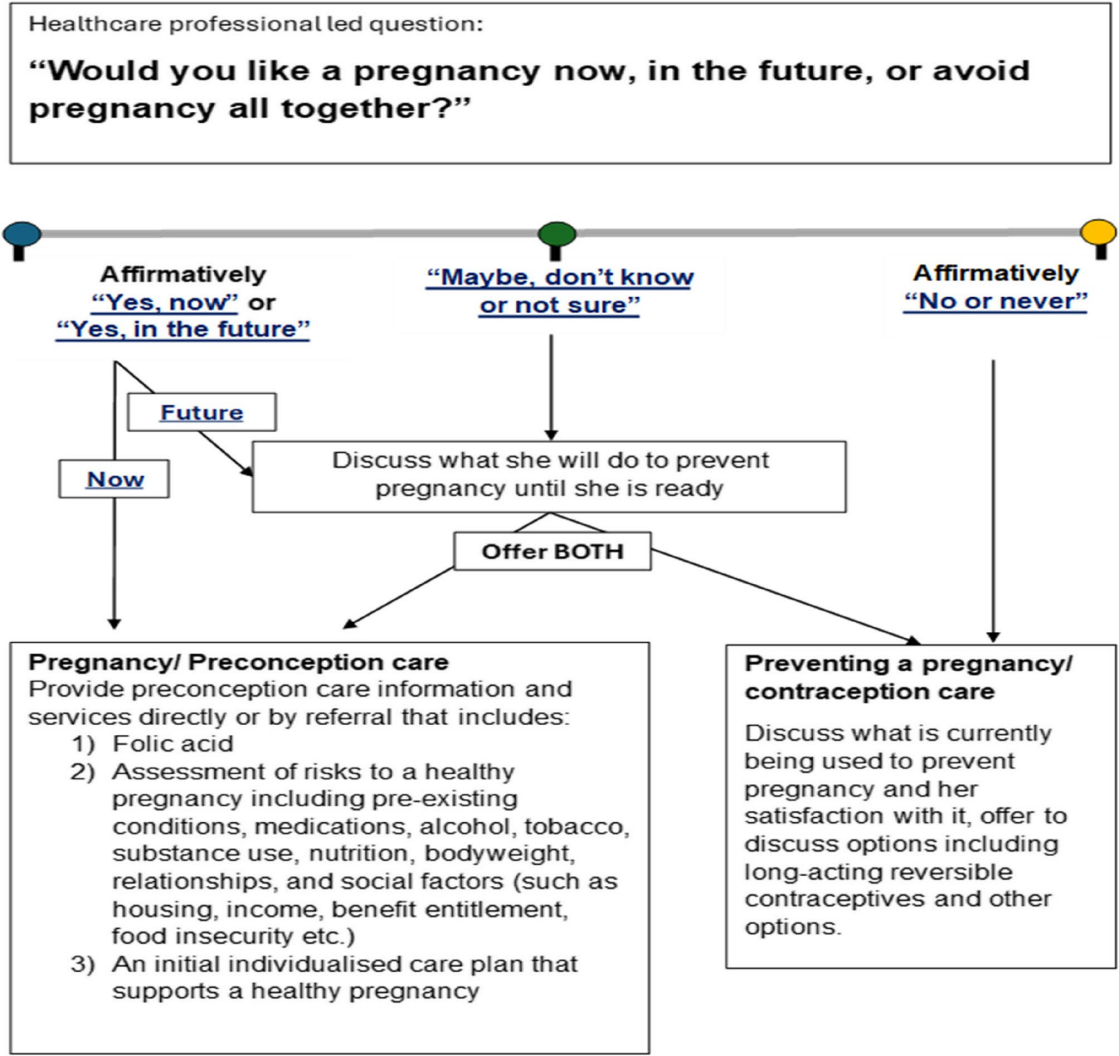
Flow diagram of the pregnancy screening tool

One of the participating clinics was a designated ‘Deep End’ practice in the East End of Glasgow, part of a network of general practices that serve populations living in the most socioeconomically deprived areas of Scotland [19] and one general practice for people experiencing homelessness within a health and social care partnership in Edinburgh. A total of ten healthcare professionals (HCPs) participated in the pilot: nine in the Edinburgh practice (including nurses and GPs) and one Advanced Nurse Practitioner in the Glasgow practice. All agreed to ask every woman of reproductive age (aged 16 and over, with the capacity to become pregnant), attending for any reason, the same pregnancy desire question: “Would you like a pregnancy now, in the future or avoid a pregnancy altogether?”. Information, services, and referrals based on whether patients answered ‘yes’, ‘maybe/don’t know/not sure’, or ‘no’ were then offered. See Fig. 1 for overview and further detail in Supplementary material 1.

### Research team

The research team co-developed the pilot in collaboration with local healthcare providers and national stakeholders, including Healthier Pregnancies, Better Lives (HPBL) working group, the Queen’s Nursing Institute Scotland (QNIS) and the Scottish Government. The same team was responsible for both supporting the implementation of the pregnancy screening tool in the participating GP practices and conducting the independent evaluation. This integrated approach ensured fidelity to the intervention design while allowing for contextual adaptation and reflective learning throughout the pilot period. Training was provided to the HCPs in the two clinics by two Queen’s Nurses who were key in driving this work. Queen’s Nurses are expert practitioners who have been nominated and awarded the title from the QNIS after completing training in clinical leadership [20].

### Study design

**Fig. 2:**
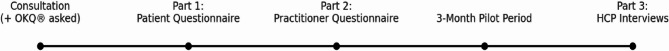
Timeline of data collection for pregnancy screening tool pilot study

The evaluation of using the pregnancy screening tool consisted of quantitative and qualitative components. The timeline of data collection is represented in Fig. 2. The study has been reported in line with the consolidated criteria for reporting qualitative research (COREQ) [21] (Supplementary material 2).

Part 1 of the evaluation was conducted by asking the patient to complete a questionnaire (Supplementary material 3), either on paper and posting it into a sealed box at the reception, or by scanning a QR code and completing the form on their device. The questionnaire was constructed on Microsoft Forms and consisted of an information sheet, consent form and eight questions. No additional demographics were collected from the patients to maintain confidentiality in this pilot study. Questionnaires were anonymised. Patients were asked by the HCP at the end of their consultation, if they would like to complete the short questionnaire, while still in the clinic or shortly after their visit. Patients were given full autonomy on whether they would like to do this or not.

Part 2 of the evaluation consisted of 4 questions and a comments section (Supplementary material 4) for the HCPs to complete immediately after the appointment where they had asked the pregnancy screening question. This was done either by scanning a QR code taking them to the Microsoft Form or by completing a paper version of the questionnaire and submitting it in the same box as the patient questionnaire. In the confidence rating scale, response options were presented as numerical values (10, 8, 5, 3, 1) with associated descriptors. The descriptor at the 3-point marker was “dissatisfied,” which was the wording used in the original questionnaire. For the purposes of analysis, this was interpreted as representing lower levels of confidence.

Part 3 of the evaluation involved interviewing the HCPs using a semi-structured interview on Microsoft Teams. This was conducted at the end of the 3-month pilot period (Fig. 2), allowing HCPs to reflect on their cumulative experiences of using the pregnancy screening tool in practice. To ensure participant anonymity, quoted responses were assigned identification numbers. EB led and recorded the HCPs interviews online using Microsoft Teams. No one else was present during these online interviews. Participants were given an information sheet to read to be able to provide informed consent. EB verbally checked for understanding of the information sheet, and consent forms were completed prior to the interviews. Before beginning any recordings, participants were made aware they could stop the interview at any time.

The separation in timing allowed patients and practitioners to provide real-time feedback on the immediate acceptability of being asked or asking the question (Parts 1 and 2), while HCPs could offer broader reflections on implementation, service delivery challenges, and outcomes after gaining experience with the tool over several weeks. Paper questionnaires from Parts 1 and 2 were periodically sent to researcher EB using *Signed For* postage.

All evaluation tools for Parts 1–3 were validated by Healthier Pregnancies, Better Lives (HPBL) working group which included a group of professionals with an interest and expertise in preconception health and care and two Queens Nursing Institute Scotland nurses. This study used an adapted version of the One Key Question^®^ (OKQ^®^) tool, originally developed by Power to Decide^®^ in the United States. The core question – ‘Would you like to become pregnant in the next year?’ – was kept. However, several adaptations were made to ensure cultural and healthcare system relevance for a Scottish population. These included: (1) using ‘Are you thinking about getting pregnant in the next year?’ to better align with local communication styles; (2) tailoring follow-up responses to reflect UK/NHS service pathways (e.g. contraceptive options, folic acid advice, or GP referral); and (3) integrating the tool into existing routine appointments rather than as a standalone intervention. These changes were developed in consultation with HPBL, QNIS Nurses, Scottish Government, Scottish healthcare professionals and public health stakeholders to support patient-centred, non-directive implementation within the local context.

Ethical approval for the evaluation of this pilot study was granted on 02/08/2024 by the Faculty of Social and Applied Sciences Research Ethics Committee at Abertay University - Ref EMS9188.

### Data analysis

Data from Part 1 (closed questions) were entered into Excel and analysed descriptively using frequency counts and percentages to summarise participant responses. This included calculating the proportion of patients who found the question appropriate, felt listened to, supported or were comfortable being asked about pregnancy intention. Participants were also asked what information they were given during the consultation (e.g. about contraception or preconception health) and whether they would have liked additional information. These responses provided further insight into the type and amount of information patients found helpful or desired.

Data from Part 2 (the healthcare professional questionnaire) were analysed using both quantitative and qualitative methods. The 4 multiple-choice questions were analysed descriptively using frequency counts to summarise practitioners’ immediate responses regarding the usability, acceptability and outcome of the pregnancy screening tool. The open-ended responses were analysed thematically using an inductive approach. These were read and re-read by the lead researcher (EB) to identify recurring patterns and insights. Initial codes were developed and grouped into broader themes reflecting practitioners’ views on the acceptability, usefulness, and practicality of asking the pregnancy intention question. This analysis was conducted manually to capture the nuanced feedback provided by HCPs following each use of the tool in practice. It is important to note that instances where the question was not asked were not systematically recorded. Healthcare professionals reported that completing a questionnaire after every patient encounter was not feasible within the constraints of a busy clinical setting. As a result, they only completed the form when they asked the question, meaning that non-use data could not be captured or quantified.

A practical thematic analysis (PTA) approach was used to analyse the interview transcripts (Part 3) since this method aligns with COREQ framework [21, 22]​. PTA was conducted in three steps. Step 1: all the transcripts were read, and short notes were written to begin to scope how these transcripts related to the research question. Step 2: the data was coded. Coding was done by hand using Excel. Researchers EB and KB developed a codebook to allow the final step (Step 3) of developing draft themes derived from the data.

## Results

### Evaluation part one - how the pregnancy desire question was perceived by women attending the clinics

A total of 56 women were asked the pregnancy intention question during the three-month pilot across two clinics. This number was lower than expected due to limited staff availability, repeat attenders (the tool was only used once per patient), and clinical judgement to omit the question in certain contexts (e.g. mental health or safeguarding concerns).

Thirty-eight women (68%) completed the evaluation questionnaire after their appointment, 21(55%) from the Glasgow Practice and 17(45%) from the Edinburgh Practice. However, not all participants answered all the questions.

Women who completed the questionnaire (participants) were asked for feedback on the HCP asking about pregnancy desire (Table 1). The vast majority said that they found it comfortable (92%), they felt listened to and supported by the practitioner (94%). A small number of participants expressed discomfort or dissatisfaction with being asked about pregnancy intention.

**Table 1:** Participant responses to practitioners asking pregnancy screening question

Question 1: Thinking about the practitioner asking you about your desire to become pregnant. Please tell us how that was for you (Please circle one for each row, 5 = most positive, 1 = most negative). Results given in *n* (%).					
	5	4	3	2	1
Easy	30 (85.7)	2 (5.7)	1 (2.9)	0 (0.0)	2 (5.7)
Helpful	29 (87.9)	2 (6.1)	1 (3.0)	0 (0.0)	1 (3.0)
Appropriate	30 (90.9)	1 (3.0)	1 (3.0)	0 (0.0)	1 (3.0)
Informative	29 (87.6)	2 (6.1)	0 (0.0)	0 (0.0)	2 (6.1)
Comfortable	34 (91.7)	0 (0.0)	2 (5.6)	0 (0.0)	1 (2.8)
Listened to	33 (94.3)	1 (2.9)	0 (0.0)	0 (0.0)	1 (2.9)
Supported	31 (93.9)	1 (3.0)	0 (0.0)	0 (0.0)	1 (3.0)

**Fig. 3:**
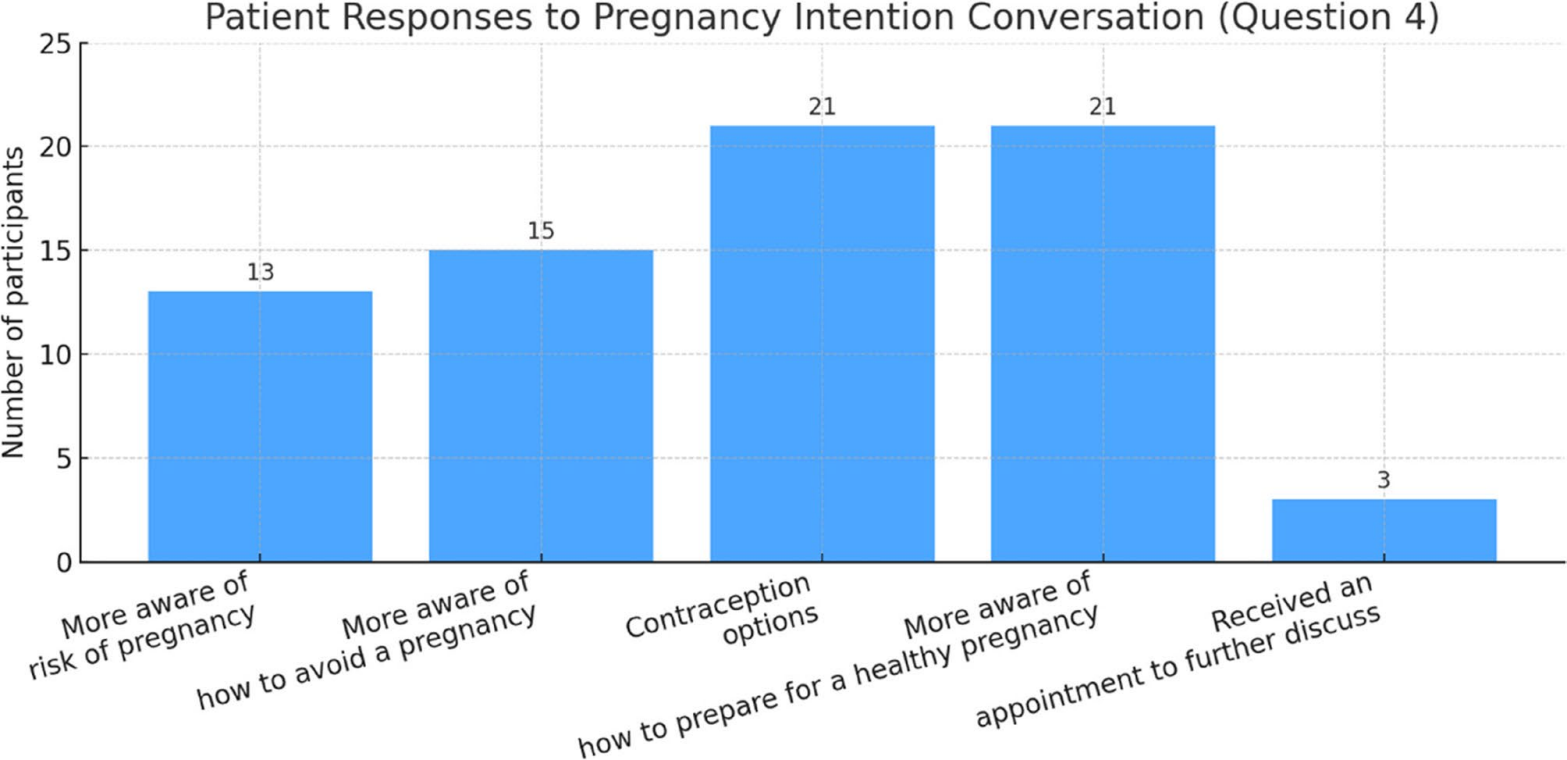
Responses to Question 4: ‘Which of the following statements describe how you feel after discussing with the practitioner about your desire to become pregnant?’ (Multiple responses permitted.)

When asked, “Had you been thinking about preventing or preparing for a pregnancy before your conversation today?”, half of the participants answered yes (*n* = 19, 50%), with the rest of them answered no (*n* = 16, 42%) or unsure (*n* = 3, 8%). Participants were asked to indicate how they felt after discussing pregnancy intention with a healthcare practitioner (Question 4 of the patient questionnaire – participants could select multiple answers). The most selected responses were “It helped me think about my contraception options” (*n* = 21) and “I am more aware of what I can do to prepare for a healthy pregnancy” (*n* = 21), as shown in Fig. 3. In addition, few participants reported being more aware of pregnancy risk (*n* = 13) or receiving an appointment to discuss further (*n* = 3).

Moreover, participants reported receiving various types of information, and some indicated that they would have liked additional support or materials (Table 2). Most of the respondents stated they were given information about how to avoid a pregnancy and contraception. Some also showed interest in knowing more about folic acid, diet and exercise.

**Table 2:** Patients’ responses to information given by HCP

Question: What information were you given today and what information would you like?		
	Information given today	More information desired
	*n* (%)	*n* (%)
How to have a healthy pregnancy	18 (75.0)	6 (25.0)
How to avoid a pregnancy	27 (93.1)	2 (6.9)
Contraception	27 (93.1)	2 (6.9)
Folic Acid	15 (62.5)	9 (32.5)
Diet	16 (66.7)	8 (33.3)
Exercise	14(63.6)	8(36.4)
Weight management	15 (68.2)	7 (31.8)
Smoking	18 (78.3)	5 (21.7)
Alcohol	17(77.3)	5 (22.7)
Other substance use	18 (75.0)	6 (25.0)
Benefit support	15 (68.2)	7 (31.8)
Parenting support	13 (65.0)	7 (35.0)
Accessing food provision	16 (69.6)	7 (30.4)

**Fig. 4:**
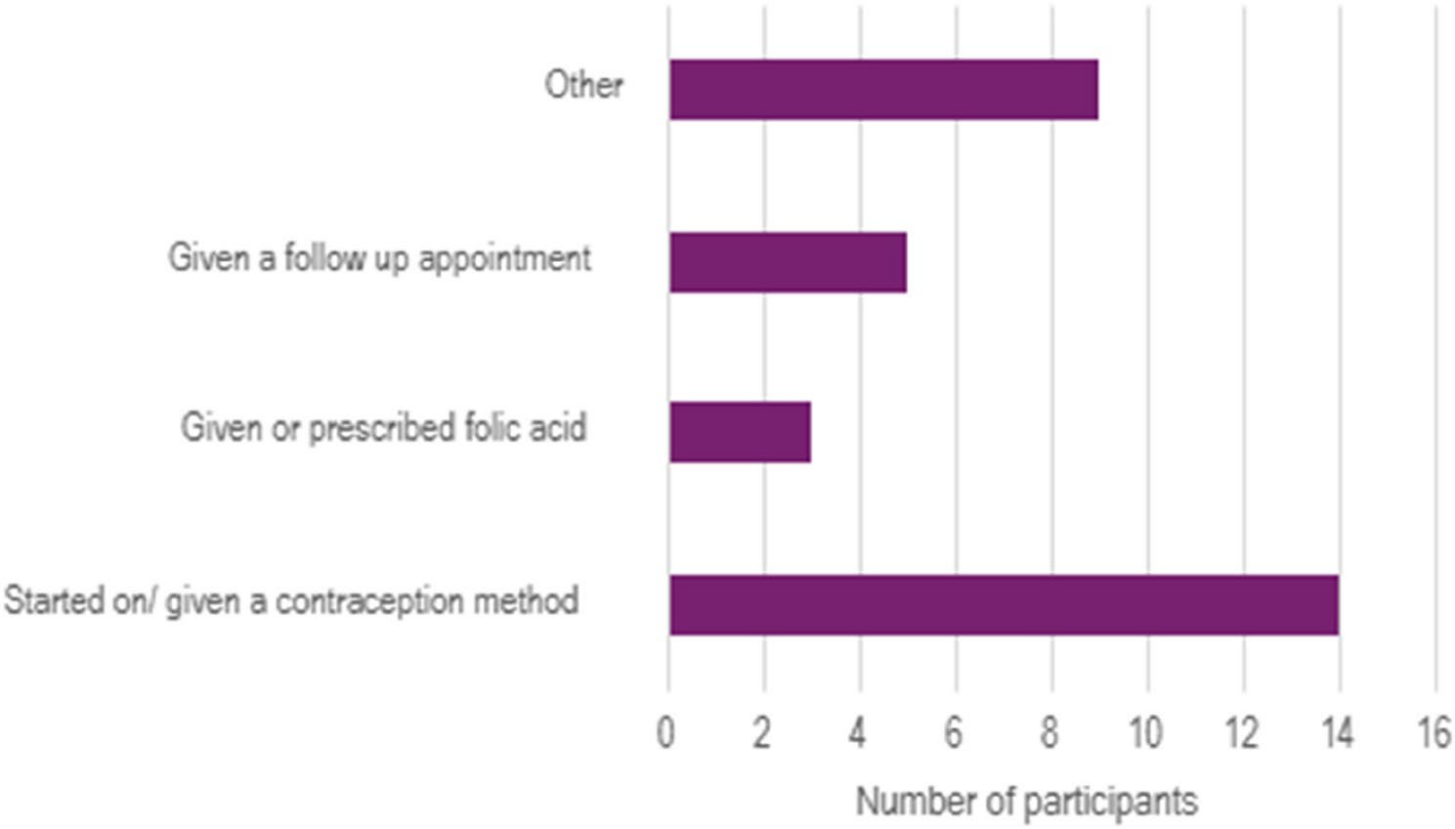
Outcome of appointment after pregnancy intention question

Just under half (*n* = 14, 45%) of the respondents who answered the question (*n* = 31) reported that during their appointment they decided to start on or were given a contraception method. The remainder were either given or prescribed folic acid (*n* = 3,10%) and/or a follow up appointment (*n* = 5,16%) (Fig. 4). Other responses (*n* = 9, 29%) included: given a pregnancy test (*n* = 1) or a leaflet (*n* = 3) or refused an intervention (*n* = 2).

The final question of the survey asked if the women felt listened to when being asked the pregnancy screening question. Many of the participants felt they were listened to about their pregnancy desire (*n* = 32, 91%), although a small number were unsure (*n* = 2, 6%) or thought they were not listened to (*n* = 1, 3%).

## Evaluation part two - service delivery issues/outcome from HCPs

Fifty-six responses were recorded by HCPs after asking the pregnancy screening question. Similarly to the patient questionnaire, HCPs did not always answer all the questions in the questionnaire. Of the 56 responses, 34(61%) came from the Edinburgh Practice and 22(39%) came from the Glasgow Practice.

The short survey was completed at the end of each appointment where pregnancy intention was asked. When asking the pregnancy desire question, over half of the HCPs reported feeling very confident, while one HCP stated not feeling confident (Table 3). The nurse in Glasgow reported feeling very confident using the tool, explaining that this confidence grew as she used it more frequently during consultations.

**Table 3:** HCPs responses in relation to their confidence when asking the question

Question: How confident did you feel asking the test of change question? 10- Very confident; 8- Confident; 5- Neutral; 3- Dissatisfied; 0- Not Confident	
Response	*n* (%)
10 - Very confident	37 (66.1)
8 - Confident	16 (28.6)
5 - Neutral	2 (3.6)
3 - Dissatisfied^a^	(0)
1 - Not confident	1 (1.8)

^a^The descriptor “dissatisfied” reflects the wording used in the original questionnaire. For analysis, this response was treated as indicating lower confidence

**Fig. 5:**
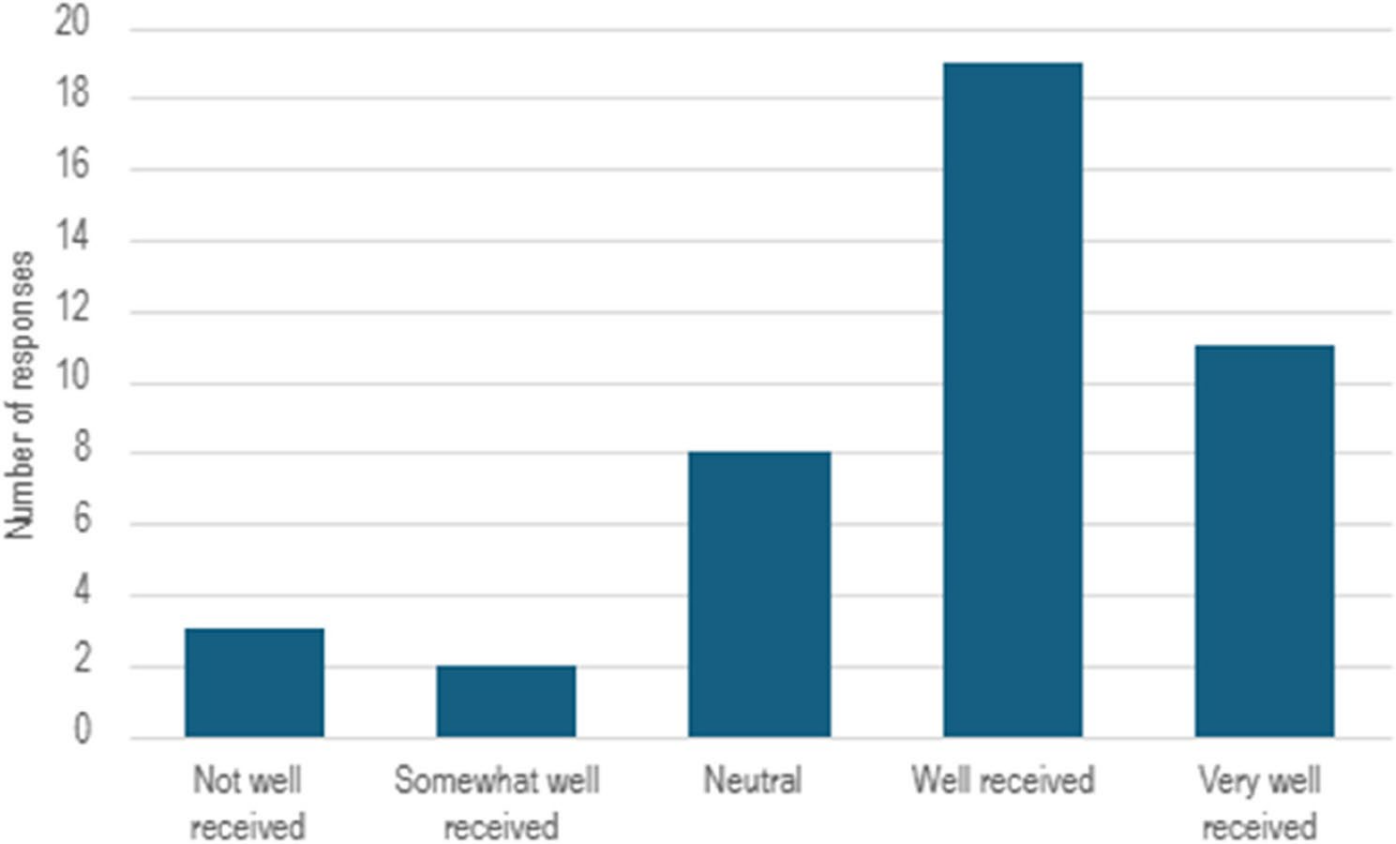
How did you feel the question was received by the patient during the consultation?

When asked how they felt the question was received by the patient during the consultation, the HCPs interpretation of how the patient responded varied (Fig. 5).

Of the 43 healthcare professional questionnaire responses, 19 (44%) and 11 (26%) indicated, respectively, that the question was well and very well received by the patient while 3 responses (7%), the HCP reported that the question was not well received.

In line with the requests for information about different topics by the participants (Table 2), the health professionals reported some actions and outcomes. The proportion of each outcome and actions taken are presented in Table 4.

**Table 4:** Outcomes and actions taken by healthcare professionals due to using the pregnancy screening question (multiple responses permitted)

Question: What were the discussion outcomes as a result of the pregnancy screening question? (Tick all that apply)	
	*n* (%)
Contraception discussion	44 (48.0)
Contraception given	12 (13.0)
Folic Acid prescribed	4 (4.0)
Referral Smoking Cessation	2 (2.0)
Referral Addiction Services	0 (0)
Initial Care Plan that supports a healthy pregnancy	1 (1.0)
Pregnancy testing	4 (4.0)
Follow up appointment nurse	9 (10.0)
Follow up appointment GP	2 (2.0)
Referral Sexual Health Clinic	1 (1.0)
Referral Weight Management	0 (0)
Risks to healthy pregnancy discussed - pre-existing conditions, medications, alcohol	12 (13.0)
Client did not want information	0 (0)

The most frequently reported outcome of asking the pregnancy screening question was contraception discussion (*n* = 44, 48%). In addition, some participants (*n* = 12, 13%) were provided with contraception during the consultation and the same number had a discussion of pregnancy-related health risks.

When asked for any other comments thirty-one responses were given. The most common being that a Nexplanon (Long-acting Reversable Contraception (LARC) was inserted or more information was given about the discussions had with patients.

### Evaluation part three- how asking the question was perceived by HCPs

**Fig. 6:**
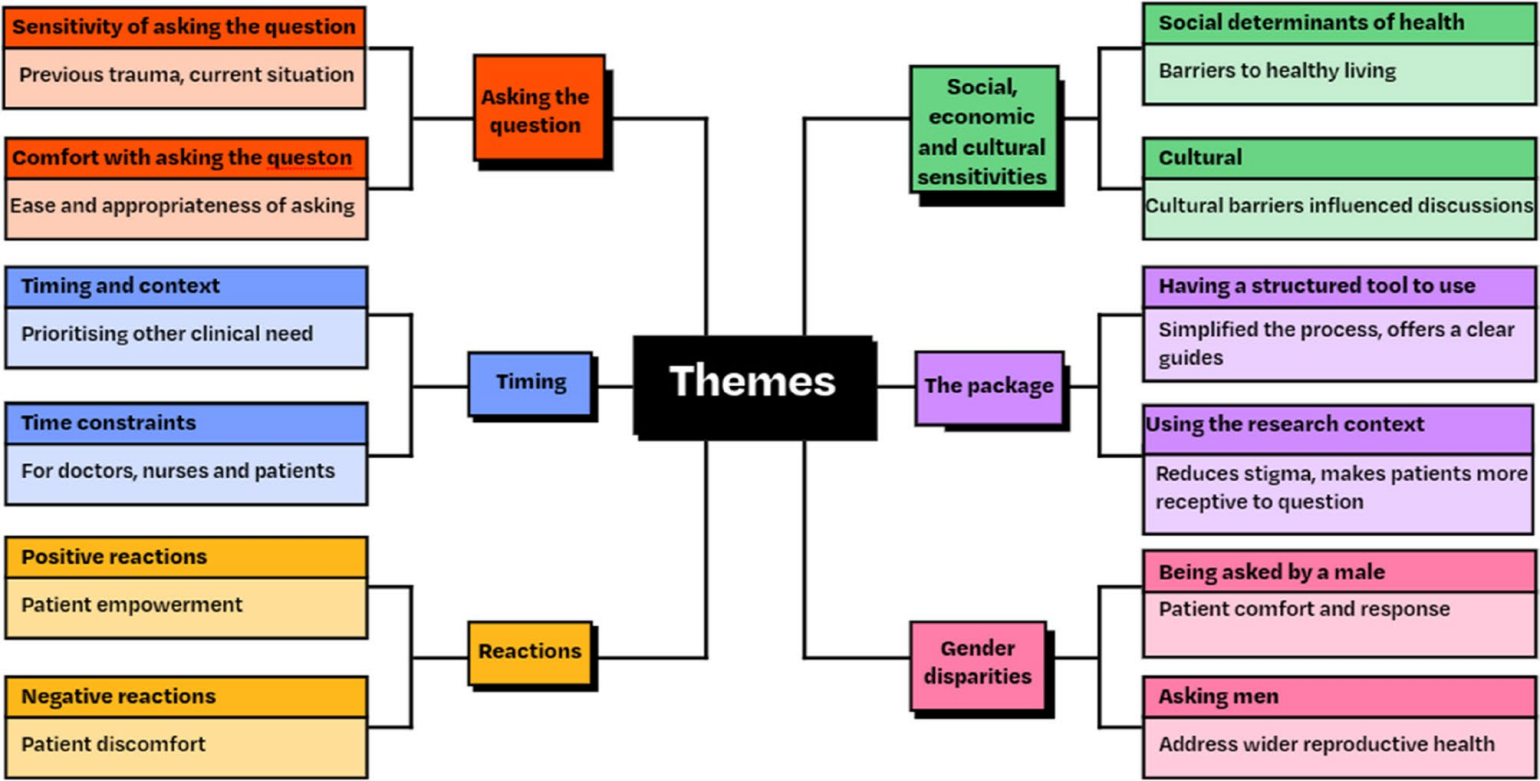
The six main key themes that emerged from the transcripts

Six HCPs were able to take part in a semi-structured one to one interview. Three of these were GP’s and three were nurses spanning the 2 practices. Interviews were conducted at the end of the 3-month pilot. Four other HCPs were invited for an interview, but due to various unavoidable reasons these could not go ahead. The duration of the interviews varied between 20 and 40 min. The key themes emerged from the transcripts were “Asking the question,” “Timing,” “Reactions”, “Social, economic and cultural sensitivities”, “The package” and “Gender disparities” (Fig. 6).

### Asking the question

The first key theme that emerged was around the sensitivity of asking the question. In general, some practitioners expressed initial discomfort with asking pregnancy intention, particularly in contexts involving previous trauma or complex social circumstances. Some initially felt the question was “not normalised” in primary care and could evoke strong reactions:

*“The person has immediately felt I was making a judgement on her ability to be a parent… it’s not a good thing to open that up in a short consultation where the agenda is really something else.”* (GP 1).

Despite this, several noted the importance of asking the question:

*“I was a very strong believer in how important it was. It did mean I then asked it to more people. I thought I was asking it to everybody.”* (GP 3).

In addition, practitioners reported that their confidence grew over time and asking the question became easier:

*“Once I’d asked it the first time, I felt quite confident… it became quite natural.”* (Nurse 2).

It should be noted, however, that at times HCPs felt the need to rephrase the question and tailor their approach, particularly for those with recent trauma or complex social histories:

*“Sometimes the question was difficult to ask, like for patients who gave birth last year and had their child taken into care. I would word it a bit differently.”* (Nurse 3).

The question was particularly relevant where pregnancy planning had clear clinical implications (e.g., prescribing teratogenic medications):

*“We had somebody prescribed drugs they should not have been conceiving on, who was trying for pregnancy and hadn’t told us… now I have the right question to ask.”* (Nurse 2).

Although recognising the importance of the question, practitioners referred the need to omit the question in situations such as acute mental health crises or when pregnancy was clinically impossible.

### Timing

Practitioners recognised the importance of the context for when the question is asked and the challenge of integrating the question into GPs consultations because of time constrains, particularly when balancing with multiple priorities:

*“It’s an important question, but in a 10-minute slot you’re already juggling a lot.”* (Nurse 3).

*“I had time to ask the question itself*,* but for detailed discussions*,* I’d say*,* ‘Let’s bring you back for another appointment.”* (Nurse 2).

They also noted the importance of introducing the question when prescribing medications, and of choosing an appropriate moment that prioritises other clinical needs where necessary:

*“Anybody who I’m prescribing medication for who is female of childbearing age… absolutely I should be asking the question.”* (Nurse 2).

It has been highlighted that certain types of appointments—such as new patient assessments, contraception consultations, or medication reviews—were particularly suitable for asking the pregnancy intention question:

*“Now that I’ve been brought to awareness*,* then it’s like when I’m asking about smears*,* contraception*,* it just will fit right into that.”* (*Nurse 1)*.

### Reactions

Patient responses to being asked the pregnancy intention question varied widely, reflecting the emotional complexity of the topic. Some patients responded with appreciation, while others expressed confusion, discomfort, or even anger:

*“One of my colleagues had somebody storm out of the room… they felt it was a rupture of the relationship.”* (GP 3).

On the other hand, positive responses often led to valuable conversations about contraception, menstrual health, menopause, or preconception care:

*“One patient said, ‘Absolutely not, I don’t want to fall pregnant,’ and she wasn’t using contraception… I put in an implant there and then.”* (Nurse 3).

Practice nurses tended to have an overall positive experience of asking about pregnancy intention.

*“It opens up conversations you otherwise wouldn’t have. If you don’t ask*,* people will present pregnant*,* and you’ve missed the boat to help them be as healthy as possible before conception.”* (*Nurse 3)*.

### Social, economic and cultural sensitivities

Cultural barriers and social determinants of health shaped discussions, with some patients showing lack of basic knowledge of preconception health.

*“It opened up conversations about vulnerabilities*,* and actually how a baby can be made*,* going back to the basics for some people.” (GP 3)*.

Cultural sensitivity also played a crucial role in how the question was received. Some patients expressed surprise—and relief—at being asked about contraception in a supportive and non-judgemental way:

*“She said*,* ‘In [country]*,* they don’t usually ask these questions. It’s all a bit taboo and private.’ She was really pleased to be asked.”* (*Nurse 3)*.

In some cases, the question enabled discussions in cultural contexts where reproductive health is seldom addressed. One particularly powerful account came from a consultation with a woman who had fled domestic violence. Initially hesitant, she became empowered by the opportunity to discuss contraception openly:

*“She said*,* ‘Do you mean I can actually talk to you about contraception?’ and I said*,* ‘Yes*,* anytime.’ She said*,* ‘Wow*,* this is OK? We can have this discussion?’ I said*,* ‘Yes*,*’ and it was… my top consultation of the year. Her revealing such a horrible situation and then actually feeling so empowered by the fact that she would have choice.”* (*GP 3)*.

### The package

HCPs valued the simplicity of the structured tool and visual aids, which offered a clear guide.

*“I had that in front of me the whole time. It’s still on my desk. I need a kind of prompt…It’s simplified*,* which is what you need. You don’t want to have big flow charts.”* (*Nurse 1)*.

They also acknowledged that providing a research context helped patients feel more open to the discussion.

#### Gender disparities

Only one of the ten clinicians was male. He noted awareness of how his gender might influence comfort or responses:

*“I’m a man asking the question… I kind of judge it case by case.”* (Nurse 1).

Practitioners noted that focusing solely on women can overlook shared decision-making:

*“…the man didn’t want more kids… then I saw his partner… she wouldn’t mind if she was pregnant. How much choice does the other potential parent have?”* (GP 1).

Conversations with men tended to focus on STIs rather than pregnancy planning:

*“…we talk to men about condom use, but it’s more from an STI perspective.”*(GP 2).

Emerging guidance, such as on Sodium Valproate, was seen as an opportunity to engage men more proactively:

*“…we need to talk to men about contraception because it’s a risk if the pregnancy has come from them.”* (GP 2).

These reflections highlight the importance of extending pregnancy intention and preconception care discussions to all individuals with reproductive capacity, regardless of gender.

## Discussion

This study evaluated the acceptability and impact of a pregnancy intention screening tool in Scottish primary care settings. Findings indicate that both healthcare professionals (HCPs) and patients generally viewed the tool as acceptable, particularly in its ability to support person-centred, timely discussions around reproductive intentions and preconception health. A key outcome was that 45% of consultations involved contraceptive discussion or provision, suggesting that the tool may serve a dual role in both pregnancy prevention and preparation.

Interviews revealed that HCPs chose not to ask the pregnancy intention question in specific contexts—such as during acute mental health crises or when they were confident, based on medical history and clinical judgement, that pregnancy was not possible. In these cases, especially where practitioners had an established longstanding relationship with the patient, this omission was often a considered and appropriated decision rather than a reflection of bias.

These findings highlight the importance of balancing universal screening with clinical judgement. While the tool was designed for routine use, practitioners valued the flexibility to tailor conversations to the patient’s context. Results suggest consistency of use could be improved to reduce the risk of missed opportunities or inequitable application.

The tool’s simplicity and the availability of visual aids, such as the flowchart, were valued by HCPs and supported integration into routine care. Asking the question often led to meaningful clinical actions, comprising initiating contraception, prescribing folic acid, or adjusting medications, all of which are key components of optimising preconception care. Some HCPs indicated they would continue using the tool in targeted consultations (e.g., new patient consultations, medication reviews or contraception discussions), showing its adaptability.

These responses suggest that even for individuals with pre-existing reproductive intentions, the screening question can enhance awareness and prompt informed action. This is particularly relevant given that preconception health is rarely discussed in clinical or everyday settings. Many patients lacked basic knowledge about folic acid, pregnancy risks, and healthy behaviours, underscoring the potential for this tool to serve an educational role within consultations. This highlights its dual function—as a prompt for reproductive health dialogue and as an intervention tool to promote health optimisation prior to pregnancy.

These findings are consistent with previous research advocating for non-judgemental, patient-centred contraceptive counselling, including during abortion care [23]​. However, HCPs reported challenges in addressing pregnancy intention within time-limited consultations, particularly when managing competing priorities or working with vulnerable populations. This reflects earlier work highlighting tensions between clinical agendas and women’s autonomy [23]​ ​. Although the tool was designed to promote autonomy, practical and systemic barriers limited its consistent application. These findings also align with Hall et al. (2024), who found that women preferred questions about pregnancy intentions to be introduced sensitively, at appropriate times, and by trusted professionals, emphasising the importance of context and relationship in these discussions [24].

Some participants in this study expressed discomfort with being asked about pregnancy intention during unrelated appointments. While this may reflect the sensitive nature of the topic, it also reiterates the importance of context, timing, and patient readiness when introducing reproductive health discussions. This reveals the need for further studies to explore patient preferences and perceived appropriateness in more detail to guide sensitive implementation.

The version of One Key Question^®^ (OKQ^®^) used in this study was adapted for the Scottish context, with permission from Power to Decide^®^, and was found to be acceptable to both patients and clinicians. Consistent with previous findings, consultation time added was minimal [25, 26]​. While some studies reported limited change on contraceptive counselling or provision following use of the OKQ^®^ tool ​ [15] ​ [27], this study observed that contraception discussions were a common outcome. However, due to the absence of baseline contraceptive data and lack of systematic documentation of who was asked or omitted, we cannot draw definitive conclusions about the tool’s direct impact on contraceptive uptake and outcomes.

A general lack of knowledge was observed among patients regarding preconception health, including folic acid supplementation and the risks associated with unplanned pregnancies. Many patients expressed interest in receiving more information, particularly those from diverse cultural backgrounds, highlighting the potential for the tool to have an educational function and to serve as a gateway to preconception health optimisation. Its integration into routine primary care—and possibly in non-clinical settings—could support more informed reproductive decision-making and improve preconception health—two key areas for public health improvement.

## Strengths and limitations

Strengths of this study include testing an adapted US-developed intervention in a new context, offering preliminary insights into its acceptability and feasibility in Scottish primary care. However, several limitations must be acknowledged. The sample size was relatively small, and there is potential for non-response bias—individuals who had negative reactions to being asked the pregnancy intention question may have chosen not to complete feedback forms. The relatively small number of women asked the question highlights real-world implementation challenges in primary care. These included limited staff capacity, contextual decisions about when to ask the question, and the fact that repeat attenders were not asked more than once, reducing the number of unique encounters in which the tool was used. A critical limitation is that the study did not capture systematic data on when or why the tool was not used. This limits our ability to assess the consistency of implementation, quantify missed opportunities, or fully understand healthcare professionals’ decision-making processes. Although interviews suggested that the question was sometimes omitted for valid clinical reasons, these were not routinely recorded. Future studies should include a method to track non-use (e.g. brief audit logs or checklists) to better assess reach and equity.

To our knowledge, this is the first study to evaluate an adapted pregnancy intention screening tool in a Scottish context. It offers valuable insights for policymakers and service providers aiming to improve reproductive health services and provides direction for refining implementation strategies to ensure equitable, effective delivery.

## Future research

Future studies should explore the optimal frequency for asking the pregnancy intention question, for example, whether it should be asked annually, once ever, or at key life stages. Further research is also needed to investigate the long-term outcomes and behavioural impacts of repeated use of the tool. To assess its true impact, future evaluations should capture baseline contraceptive use and include mechanisms to monitor when and why the tool is not used. Similarly, it will be useful to further investigate patient preferences and the perceived appropriateness of being asked this question during various types of consultations. Finally, there is a need to assess the tool’s educational potential, particularly in improving preconception health knowledge among underserved populations.

## Conclusion

This study demonstrates the acceptability of a structured pregnancy intention screening tool in Scottish primary care and its potential to support timely, person-centred discussions about reproductive goals. By enabling open, non-judgemental conversations, the tool may help women make informed decisions about pregnancy planning and contraception. While the intervention shows promise for integrating reproductive health more routinely into primary care, further research is needed to assess its broader impact, optimal implementation, and long-term outcomes across diverse populations.

## Supplementary Information

Supplementary Material 1.

Supplementary Material 2.

Supplementary Material 3.

Supplementary Material 4.

## Data Availability

The datasets used and/or analysed during the current study are available from the corresponding author on reasonable request.
