# Supplementary material for: “It’s not about a question, it’s about the outcomes, isn’t it?”: pilot study for Scottish pregnancy screening tool provision of preconception health care in Scotland
Source: Reprod Health. 2025 Nov 21;22:260. doi: 10.1186/s12978-025-02191-y (PMC12751275; doi:10.1186/s12978-025-02191-y)
Supplement: Supplementary file 1 — Supplementary Material 1. [file 12978_2025_2191_MOESM1_ESM.docx]

**Asking the pregnancy desire question**

**Question: “Would you like a pregnancy now, in the future, or avoid pregnancy all together?”**

The question may be prefaced by:

“*We are asking all our reproductive aged patients/clients (aged 16 and over) who can become pregnant, some questions about their pregnancy desire. You can answer* ***yes*** *(now or in the future),* ***maybe/don’t know/not sure, or no****. Would you like to become pregnant now, in the future or avoid a pregnancy all together?”*

Depending on her response the clinician will provide appropriate pre/inter-conception and/or contraception information and services that align with her pregnancy desire.

**“Yes, now”**

If the woman answers “**yes, now”**, it’s an opportunity to **educate her about preparing for pregnancy/preconception care.** Suggested response to encourage engagement:

“*Great. We know about what causes risks and how to prevent the risk from occurring, or at least reduce the risk for you and a developing pregnancy. If you agree, I’d like to talk with you about the biggest and most preventable risks you could face if you were to become pregnant?*”

- Provide preconception care information and services directly or by referral that includes:

1. Recommendation of and, if possible, dispense appropriate level of folic acid supplement
2. Assessment of risks to a healthy pregnancy including pre-existing conditions, medications, alcohol, tobacco, substance use, nutrition, bodyweight, relationships, and social factors (such as housing, income, benefit entitlement, food insecurity etc.)
3. An initial individualised care plan that supports a healthy pregnancy

- Establish a timeline for preconception care by asking:

“*How long would you like to wait until you become pregnant?*”

This allows you to focus your consultation on her current pregnancy desire and concerns.

If the woman has recently had a baby, and would like to have another baby soon, let her know that their chances of getting pregnant again will be boosted if both her and her partner are in the best of health. This includes giving her body time to recover from her recent pregnancy. Women are advised to wait at least 18 months before conceiving again. This will mean there is less chance of complications and health issues during your pregnancy with another baby.

- If pregnancy is not desired immediately, please follow information in next section.
- If it is not possible to offer information and services, a referral should be made to another practitioner or service that can offer preconception or inter-conception care information.

**“Yes, sometime in the future”**

If the woman answers “**yes, I don’t know when but not now”**, it is an opportunity to **educate her about both preparing for pregnancy/preconception care and preventing a pregnancy/contraception care.**

- Establish the risk of pregnancy by asking if she is currently or sometimes sexually active*.*
- If “yes”*,* ask if she has been using any contraceptive methods or taken any other actions to prevent a pregnancy. Offer contraception information or counselling if she answers no or indicates she would like a method more effective in preventing pregnancy. A prompt might be:

*“Are you doing anything to prevent pregnancy?*

- If she answers “no” or “sometimes”, give information about the risk of becoming pregnant and invite a discussion about contraception, for example:

“*Is it OK with you if we talk about different contraceptives available? What’s most important to you regarding contraception: longer or shorter acting contraception, hormone based or no hormones, having a period, not having a period, effectiveness, privacy?”*

“*Is it OK with you if we talk about different long- acting contraceptives?”*

- Invite a conversation about preconception care given she may want a pregnancy in future (see above section):

“*Since you may want a pregnancy in the future, is it OK with you if we talk about the different ways necessary to prepare for a healthy pregnancy?”*

***Further notes to support preparation for a healthy pregnancy - if woman answers “Yes, now” or “Yes, sometime in the future”***

- Most women don’t know they’re pregnant for weeks. It’s critical to support developing fetal brain health so if there is a likelihood of her becoming pregnant, recommend an appropriate level of folic acid supplement. There is no harm to the woman if she takes folic acid and does not become pregnant. Folic Acid is only contraindicated if the woman is diagnosed with pernicious anaemia and/or malignancy. A vitamin D supplement is recommended for all pregnant women, especially those with higher risk of low vitamin D. It may be worth recommending starting to take a vitamin D supplement before she becomes pregnant.

- It is also important to help the woman understand that behaviours like smoking, drinking or using street drugs can also affect the baby’s brain development.

“*It’s important not to wait until you’re pregnant to act on this”.*

- Discuss weight loss if the woman is overweight or obese. Women who are overweight or obese have more difficulty conceiving, and they are also more likely to experience complications in pregnancy such as gestational diabetes, high blood pressure, blood clots, needing a c-section, premature deliveries, and abnormalities. Practitioner can offer support to the woman to try reducing weight.

- Refer to her medication list. If she is taking medication contraindicated in pregnancy, assist her with safely stopping medication, reducing dosage, or switching to medications with fewer side effects. Many treatments for epilepsy, diabetes, high cholesterol, high blood pressure and for mental health conditions shouldn’t be taken during pregnancy. Consider whether there are treatment approaches to manage the woman’s health condition that may be more appropriate in pregnancy. Refer woman to GP or specialist for medication management as needed.

- Check the woman’s smear status. As a smear cannot be done during pregnancy, she should be advised to have a smear before becoming pregnant if she is due a smear.

- Check the woman’s psycho-social status and her wellbeing. If she is in a relationship, does she feel safe? Is her pregnancy desire her own desire or is there any coercive control?

**“Maybe/don’t know/not sure”**

If she answers “**maybe/don’t know/not sure”**, it’s an opportunity to **educate her about both preparing for pregnancy/preconception care and preventing a pregnancy/contraception care.**

- The conversation with the woman will be similar to ‘Yes, but not now’, to establish the risk of pregnancy and to invite a conversation about both contraception and preconception care given she may want a pregnancy in future.

- It is useful to note that women who express ***ambivalence*** about pregnancy may be less aware of their risk of becoming pregnant and not using an effective form of contraception. They are less likely to be prepared for a pregnancy (see ‘[***Further notes to support preparation for a healthy pregnancy***](bookmark://further_notes)*’)*. It may be helpful to note to the woman:

*“Many women don’t use contraception because they think they can’t get pregnant. 85% of those sexually active with some frequency will become pregnant within a year”.*

*“Since there’s a possibility you will become pregnant, would you like to discuss some simple ways to prepare for a healthy pregnancy.”*

- The HCPs job is **not** to resolve the ambivalence but to identify it and educate about both preconception and contraception.

**“No/Never”**

If she answers “**No/Never”**, it’s an opportunity to **educate her about preventing a pregnancy/contraception care.**

- Establish the risk of pregnancy by asking if she is currently or sometimes sexually active*.*
- If “yes”*,* ask if she has been using contraception or taken any other actions to prevent a pregnancy. Provide contraception information or counselling if she answers no or indicates she would like a method more effective in preventing pregnancy.

*“Are you doing anything to prevent pregnancy?*

- If “no” or “sometimes”, give information about the risk of becoming pregnant and invite a conversation about contraception.

“*Is it OK with you if we talk about different contraceptives available? What’s most important to you regarding contraception: longer or shorter acting contraception, hormone based or no hormones, having a period, not having a period, effectiveness, privacy?”*

- Information about the most effective long-acting reversible contraceptive methods (LARC) or permanent methods may be appropriately introduced if the woman says that she never wants to become pregnant.

*“You may be more interested in long-acting contraceptives, would you like to discuss those?”*

*“At some point, you may want to consider permanent prevention options available”.*

**Recommendations for Practitioners**

Three sets of actions can make a significant difference in helping women prepare for a safe pregnancy and a healthy baby:

What to **stop,** what to think about and **discuss** and what to **start**.

**STOP**

| 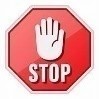 | Overtly or implicitly expressing unconscious bias that blames and shames women for the health status and behaviours – since this is not only ineffective and unkind, but also fails to address the root causes of their problems (e.g. substance abuse as a coping mechanism and self-medicating for persistent distress.) |
| --- | --- |
| 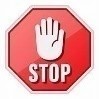 | Allowing mixed, confusing or contradictory pre/interconception care messages and advice from different members of your own staff, as well as from those to whom you refer your patient/client. |
|  | Assuming that women with a certain level of education and income have no (or only minor) preconception health problems or needs that should be explored and addressed |
|  | Prescribing teratogenic medications to women who say “Yes”, “Maybe/Don’t know”. Giving priority to physical health needs, while downplaying mental/emotional health needs. |

**DISCUSS**

| 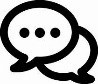 | Preconception health, education and care with the prospective fathers/partners, if that is acceptable to the prospective mother. |
| --- | --- |
|  | Where to get needed help or support for prospective parents when you cannot offer it directly. |
|  | With your staff how to present and talk about preconception health risks with patients/clients in ways that are accurate and helpful, rather than overestimating them (and creating fear) or underestimating these risks (and encouraging false confidence). |
|  | How to boost your, and your staff’s knowledge base about preconception health, education and care. |
|  | The ways in which preconception education, screening, counselling and care can best become part of the normal, routine operations of your health center centre or professional practice. |

**START**

|  | Making sure that any immunisations the woman has had in the past are still providing protection or whether a booster (e.g. Rubella) is needed, and, whether there are others that have now been recommended such as whooping cough vaccine. |
| --- | --- |
|  | Supporting each woman to take the right steps, at the right time, in the right ways to make her own reproductive goals a reality – from taking appropriate folic acid supplements at least three months prior to, and three months after, conception and suggesting that the woman could continue effective contraception while choosing to address any major risk factors identified prior to attempting to conceive. It is the woman’s choice. |
|  | Offering advice and assistance from the positive perspective that each woman already deeply desires the main outcomes that you also want for her, namely, a safe pregnancy, a thriving baby and a rewarding parental experience – as that positive attitude is essential in building or maintaining a relationship of mutual respect, trust and two-way communication. |

                                                                                                              (Source: ​(Sher. J, 2016)​
