# Supplementary material for: “It’s not about a question, it’s about the outcomes, isn’t it?”: pilot study for Scottish pregnancy screening tool provision of preconception health care in Scotland
Source: Reprod Health. 2025 Nov 21;22:260. doi: 10.1186/s12978-025-02191-y (PMC12751275; doi:10.1186/s12978-025-02191-y)
Supplement: Supplementary file 2 — Supplementary Material 2. [file 12978_2025_2191_MOESM2_ESM.docx]

**One key Question Scotland- women's responses**

We would appreciate your feedback about the service you received today which will enable us to learn what we are doing well and to identify areas for improvement. We do not ask for your personal details in this survey, so any response is anonymous and confidential.

Your participation is voluntary, it is your choice whether you take part, you can withdraw at any time and this will not affect the care you receive. What you tell us will not be linked to your records and will only be seen by the evaluation team. Your responses will assist us in the development of services in this area and the reporting of the results will be completely anonymous. The survey can be completed in the waiting room and returned anonymously to the box at reception. If you have any questions about this evaluation survey, please contact Emma Brough e.brough1700@abertay.ac.uk

**1. I understand the contents of the participant information sheet and consent form and agree to take part in this short survey**

Yes

No

**2.Thinking about the practitioner asking you about your desire to become pregnant. Please tell us how that was for you**

**(Please circle one for each row, 5= most positive, 1 = most negative)**

|  | *5* | *4* | *3* | *2* | *1* |
| --- | --- | --- | --- | --- | --- |
| *Easy* |  |  |  |  |  |
| *Helpful* |  |  |  |  |  |
| *Appropriate* |  |  |  |  |  |
| *Informative* |  |  |  |  |  |
| *Comfortable* |  |  |  |  |  |
| *Listened to* |  |  |  |  |  |
| *Supported* |  |  |  |  |  |

**3.Had you been thinking about preventing or preparing for pregnancy before your conversation today?**

Yes

No

Unsure

**4.Which of the following statements describe how you feel after discussing with the practitioner about your desire to become pregnant? (Tick all they apply)**

I am more aware of my risk of pregnancy

I am more aware of what I can do to avoid a pregnancy

I am more aware of what I can do to prepare for a healthy pregnancy

It helped me think about my contraception options

I got an appointment to discuss things further about my desire to become pregnant

**5. What information were you given today and what information would you like?**

|  | *I was given this information today* | *I would like more information about this* |
| --- | --- | --- |
| *How to have a healthy pregnancy* |  |  |
| *How to avoid a pregnancy* |  |  |
| *Contraception* |  |  |
| *Folic Acid* |  |  |
| *Diet* |  |  |
| *Exercise* |  |  |
| *Weight management* |  |  |
| *smoking* |  |  |
| *alcohol* |  |  |
| *Other substance use* |  |  |
| *Benefit support* |  |  |
| *Parenting support* |  |  |
| *Accessing food provision* |  |  |

**6. Is there anything else you would like more information about? (if yes, please write below)**

**7. Which of the following were you provided with today? (tick all that apply)**

A contraception method was given/started

Folic acid (or a prescription for) was given

A follow up appointment

**8.Did you feel listened to today, about your desire to become pregnant?**

Yes

No

Unsure
