# Supplementary material for: “It’s not about a question, it’s about the outcomes, isn’t it?”: pilot study for Scottish pregnancy screening tool provision of preconception health care in Scotland
Source: Reprod Health. 2025 Nov 21;22:260. doi: 10.1186/s12978-025-02191-y (PMC12751275; doi:10.1186/s12978-025-02191-y)
Supplement: Supplementary file 3 — Supplementary Material 3. [file 12978_2025_2191_MOESM3_ESM.docx]

**Post appointment form for Staff- One Key Question Scotland**

Date:

**1.How confident did you feel asking the test of change question?**

|  | 10- Very confident | Confident | 5- Neutral | Dissatisfied | 0- Not Confident |
| --- | --- | --- | --- | --- | --- |
|  |  |  |  |  |  |

**2.How did you feel the question was received by patient during the consultation?**

Not well received

Somewhat well received

Neutral

Well received

Very well received

**3.What were the discussion outcomes as a result of the test of change question?  (Please choose as many as applicable)**

Contraception discussion

Contraception given

Folic Acid prescribed

Referral Smoking Cessation

Referral Addiction Services

Initial Care Plan that supports a healthy pregnancy

Pregnancy testing

Follow up appointment nurse

Follow up appointment GP

Referral Sexual Health Clinic

Referral Weight Management

Risks to healthy pregnancy discussed - pre-existing conditions, medications, alcohol, tobacco, substance use, nutrition, bodyweight relationships, and social factors (such as housing, income, benefit entitlement, food insecurity etc.)

Client did not want information

**4.Comments**

Submit


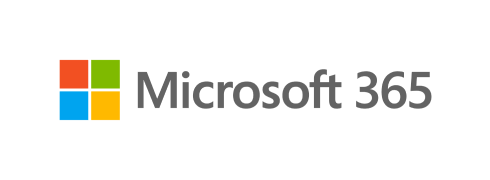


This content is created by the owner of the form. The data you submit will be sent to the form owner. Microsoft is not responsible for the privacy or security practices of its customers, including those of this form owner. Never give out your password.

Microsoft Forms | AI-Powered surveys, quizzes and polls Create my own form

The owner of this form has not provided a privacy statement as to how they will use your response data. Do not provide personal or sensitive information. | [Terms of use](https://go.microsoft.com/fwlink/?linkid=866263)
